# Intrinsically microporous polymer (PIM-1) enhanced degradation of heptadecafluoro-1-nonanol at graphitic carbon nitride (g-C_3_N_4_)

**DOI:** 10.1039/d5ra07284k

**Published:** 2026-01-02

**Authors:** Fernanda C. O. L. Martins, Wanessa R. Melchert, Akalya Karunakaran, Chris R. Bowen, Nicholas Garrod, Philip J. Fletcher, Mariolino Carta, Dominic Taylor, Neil B. McKeown, Frank Marken

**Affiliations:** a Department of Chemistry, University of Bath Claverton Down Bath BA2 7AY UK f.marken@bath.ac.uk; b Center for Nuclear Energy in Agriculture, University of São Paulo P. O. Box 96 Piracicaba SP 13400-970 Brazil; c College of Agriculture Luiz de Queiroz, University of São Paulo P. O. Box 9 Piracicaba SP 13418-970 Brazil; d Department of Mechanical Engineering, University of Bath BA2 7AY Bath UK; e University of Bath, Imaging Facility Bath BA2 7AY UK; f Department of Chemistry, Swansea University, College of Science Grove Building, Singleton Park Swansea SA2 8PP UK; g EaStCHEM, School of Chemistry, University of Edinburgh Joseph Black Building, David Brewster Road Edinburgh Scotland EH9 3JF UK

## Abstract

The photochemical transformation of polyfluorinated alkyl substances (PFAS) leads to structural unzipping to give rise to fluoride and further degradation products depending on (i) the type of photocatalyst as well as on (ii) microporous coatings or reaction environments. Here, a substantial increase in photocatalyst performance is observed by coating graphitic carbon nitride (g-C_3_N_4_) with an intrinsically microporous polymer (PIM-1) to enhance interaction with heptadecafluoro-1-nonanol (as a PFAS model).

## Abstract 2

Intrinsically microporous host for photocatalysts enhances performance and selectivity in photo-PFAS degradation at g-C_3_N_4_.

## Graphical abstract

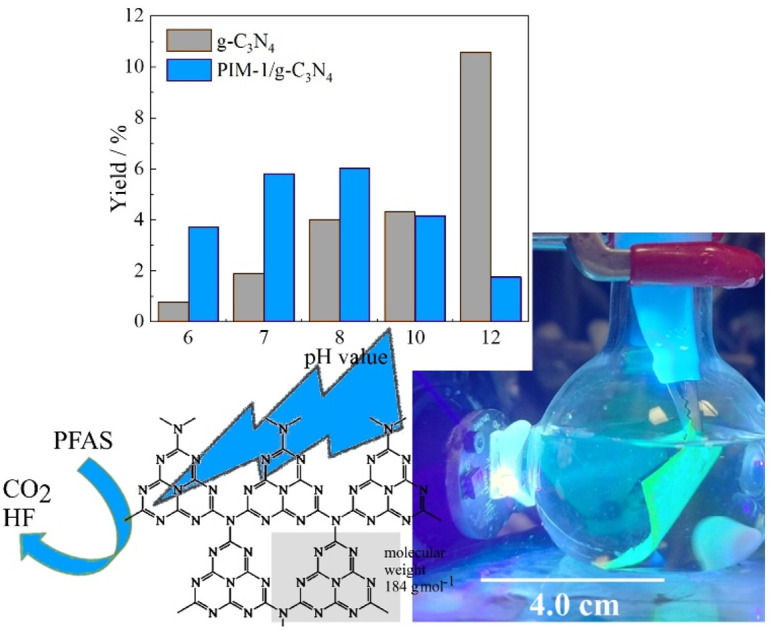

For several years, polyfluorinated alkyl substances (PFAS) have been desirable as components in consumer products, or technical products such as fire-fighting foams.^1,2^ As a result, they are now wide-spread and present as a variety of molecular types that are distributed in our environment with exceptionally slow natural degradation rates^3^ and with the potential to harm living organisms.^4^ Remediation approaches are currently based on chemical/electrochemical processes,^5^ heterogeneous catalysis,^6^ adsorption into porous carbons or metal–organic frameworks (MOFs),^7^ bio-degradation,^8^ or photodegradation with suitable photocatalysts.^9^ The photodegradation of PFAS materials has been studied primarily with metal-containing photocatalysts,^10^ but recently also with a polymer-modified photocatalyst based on graphitic carbon nitride (g-C_3_N_4_, Fig. 1A).^11^ We have prepared and employed g-C_3_N_4_ previously for example in photo-hydrogen production.^12^

**Fig. 1:**
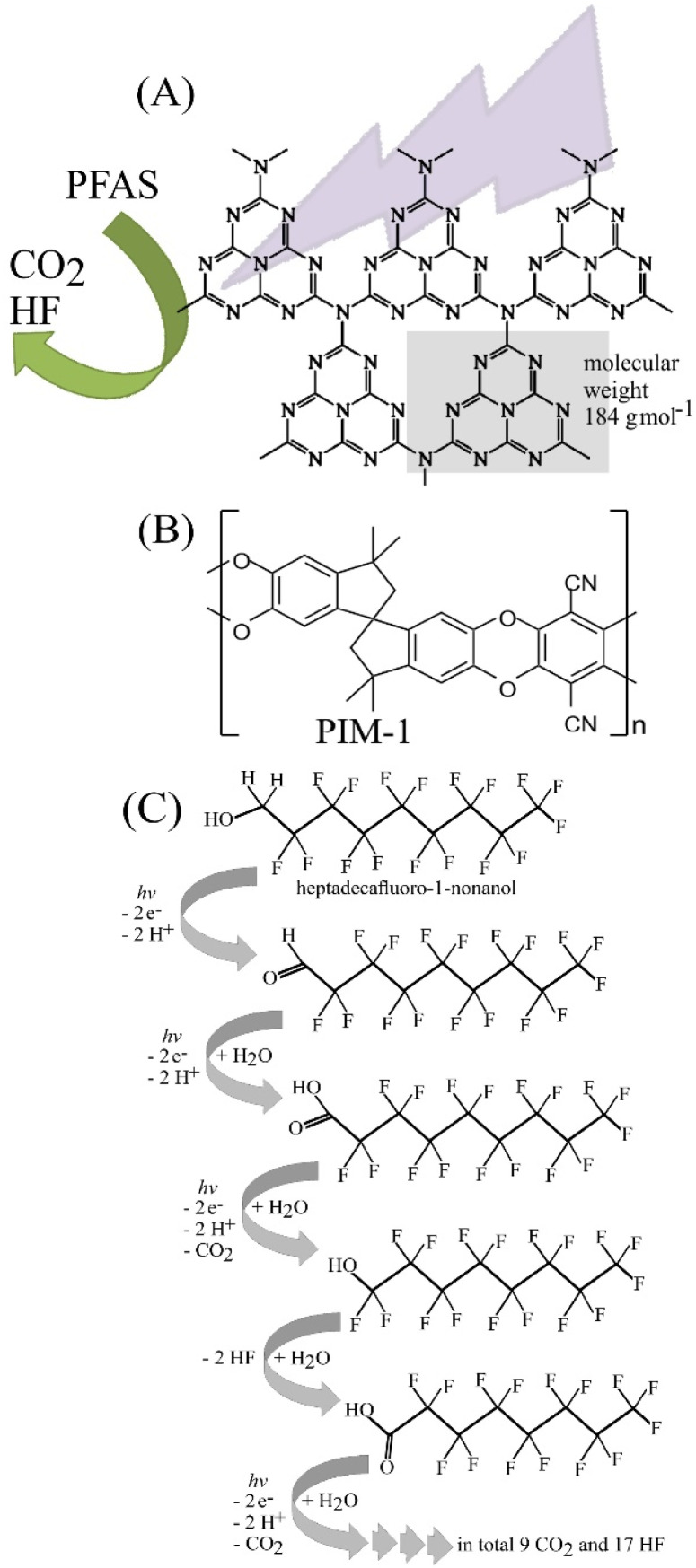
Scheme for (A) the g-C_3_N_4_ photocatalyst, (B) the molecular structure of the polymer of intrinsic microporosity (PIM-1), and (C) the step-by-step photocatalytic degradation of heptadecafluoro-1-nonanol (HDFN) into CO_2_ and HF.

For the study of photodegradation processes both the photocatalyst and the mechanism usually take centre stage,^10^ however, the surroundings of the photocatalytically active site also play an essential role. For example, intrinsically microporous host materials can beneficially affect the reactivity at the surface of photocatalysts.^13,14^ In particular, molecular rigidity in these polymer structures can affect the local concentration and reactivity of reactants without the polymers being degraded themselves.

PFAS molecules degrade *via* fluoride formation/hydrolysis. Processes based on photocatalysis,^15,16^ sorption or chemical catalysis,^17^ as well as electrocatalysis^18^ have been proposed. Here, heptadecafluoro-1-nonanol (HDFN) has been employed as a model PFAS molecule to explore degradation *via* surface-modified photocatalysts. HDFN has been reported previously as a surfactant^19^ and as a reagent in polymer surface modification.^20^ HDFN degradation in the atmosphere, as a result of attack by ^.^OH radicals, has been investigated.^21^ The hypothetical overall reaction mechanism is given in eqn (1), however, there are many possible intermediates and pathways (as well as follow up reactions with necessarily co-generated H_2_O_2_ as has been reported previously^13^) before full degradation occurs.

1 C_9_H_3_OF_17_ + 9 O_2_ + 17 H_2_O → 9 CO_2_ + 17 HF + 9 H_2_O_2_

Each molecule of HDFN ultimately results in 17 equivalents of fluoride and a fluoride selective potentiometric probe can be employed to follow the degradation process quantitatively. A commercial fluoride-selective potentiometric probe is employed here to follow fluoride production (see Experimental††Experimental. Heptadecafluorononanol (446823, Sigma-Aldrich, 2,2,3,3,4,4,5,5,6,6,7,7,8,8,9,9,9-heptadecafluoro-1-nonanol) and all other reagents were purchased and used without further purification. Polymers of intrinsic microporosity were synthesised with molecular weight > 70 kD following literature methods for PIM-1 (ref. 29) and for PIM-EA-TB.^30^ The photocatalyst g-C_3_N_4_ was synthesized using 5 g of melamine within a ceramic boat with a lid, which was placed into a tube furnace with a temperature ramp to 500 °C, where the temperature was maintained for 4 h.^31^ Instrumentation. Fluoride release was quantified with a potentiometric fluoride probe FC301B (Hanna Instruments, US). Chronopotentiometric analyses were performed with a potentiostat/galvanostat from Metrohm-Eco Chemie model µAUTOLB III with NOVA 2.1.2 software (Metrohm-Eco Chemie, NL). Zero current potentiometry was performed *versus* a saturated calomel electrode (SCE). For pH measurements, a commercial glass membrane pH-probe (Voltcraft 127752) was employed. Photochemical processes were performed with a light emitting diode (LED) light source (*λ* = 385 nm, approx. 27 mW cm^−2^ at 4 cm distance, Thorlabs Ltd). A water purification system from CE Instruments Ltd was used to obtain purified water with resistivity not lower than 18.2 W cm at 20 °C. Scanning electron microscopy (SEM) and energy-dispersive X-ray spectroscopy (EDS) was carried out on using a Hitachi SU3900 system with an Oxford Instruments X-Max 170 mm^2^ EDS detector.).

Polymers of intrinsic microporosity (PIMs^22^) have been developed mainly for applications in gas adsorption and separation;^23^ however, due to a pore size of typically 1 nm and good processability, this material and similar PIMs have found applications in a wider range of areas. The PIM-1 material (Fig. 1B) has been originally developed by Budd and McKeown^24^ and has now been employed in energy storage processes,^25^ electrochemical devices,^26^ and in photocatalysis.^27^

In this communication, the effect of intrinsically microporous polymers, namely PIM-EA-TB and PIM-1, on the photodegradation of heptadecafluoro-1-nonanol (HDFN) is reported. It is suggested that the more hydrophobic molecularly rigid polymer PIM-1 aids in the local accumulation of HDFN and thereby enhances the rate of photocatalytic fluoride formation even in the presence of a neutral phosphate buffer media (pH 7). Essentially, photodegradation occurs by coupling photo-anodic HDFN degradation with photo-cathodic oxygen reduction to H_2_O_2_ (eqn (1)) and is controlled by/within rigid micropores at the catalyst surface.

Photochemical reactions were performed in a 20 mL reactor with agitation either with a suspended photocatalyst or with photocatalyst immobilised onto a filter paper.^28^ A blue LED light was turned on (Fig. 2A) with the photocatalyst in 20 mL of 0.10 mol L^−1^ phosphate buffer solution at pH 7. A filter paper (Whatman 1; area 2 × 2 cm^2^) was modified with either (Fig. 2B) 5.0 mg g-C_3_N_4_ and 1.0 mg PIM-1, or (Fig. 2C) 10.0 mg g-C_3_N_4_ and 1.0 mg PIM-1, or (Fig. 2D) 25.0 mg g-C_3_N_4_ and 1.0 mg PIM-1, or (Fig. 2E) 50.0 mg g-C_3_N_4_ and 1.0 mg PIM-1. Coatings were prepared by drop casting with solutions in CHCl_3_.

**Fig. 2:**
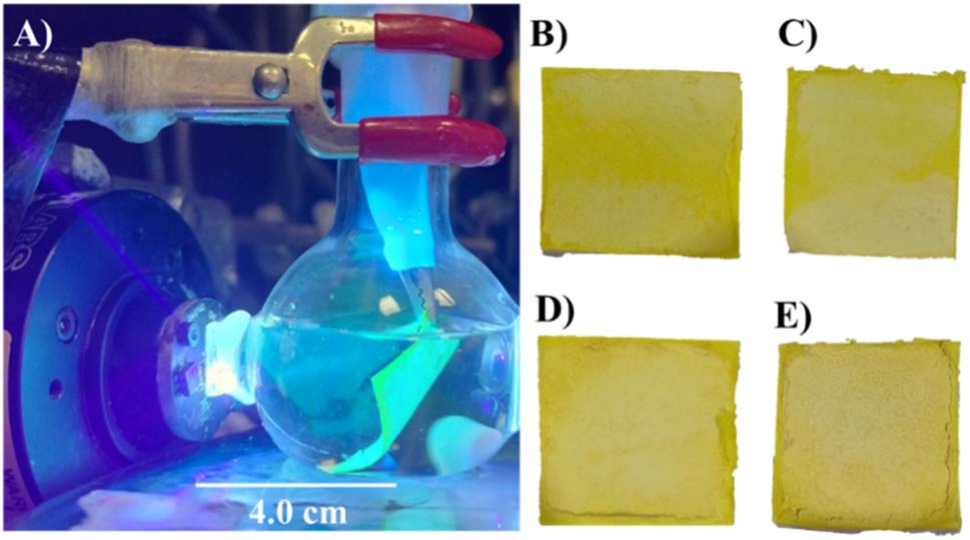
(A) Blue light turned on with filter paper modified with PIM-1 and g-C_3_N_4_ in 20 mL of 0.10 mol per L phosphate buffer solution at pH 7. (B–D) Photographs of filter paper (area 2 × 2 cm^2^) modified with (B) 5.0 mg g-C_3_N_4_ and 1.0 mg PIM-1, (C) 10.0 mg g-C_3_N_4_ and 1.0 mg PIM-1, (D) 25.0 mg g-C_3_N_4_ and 1.0 mg PIM-1, and (E) 50.0 mg g-C_3_N_4_ and 1.0 mg PIM-1.

Fig. 3 shows typical electron microscopy images for the g-C_3_N_4_ photocatalysts (flaky particles ranging in size from 0.5 to 15 µm, consistent with previous reports^12^) with/without PIM-1 on silicon or on filter paper substrates. Micropores in PIM-1 are too small (typically 1 nm) to be directly observed in SEM images. In the g-C_3_N_4_/PIM-1 composite, the photocatalyst is proposed to retain full reactivity (as reported previously^13^) due to the rigid molecular backbone of PIM-1 preventing capping/blocking of the catalyst particle surface. However, the transport of reagents and products into and out of the micropores of PIM-1 is likely to be slow. It is likely that only a surface layer of the g-C_3_N_4_/PIM-1 composite is active in photocatalysis and deeper regions might not contribute. Microporous PIM-1 can still beneficially affect the catalyst performance (*vide infra*).

**Fig. 3:**
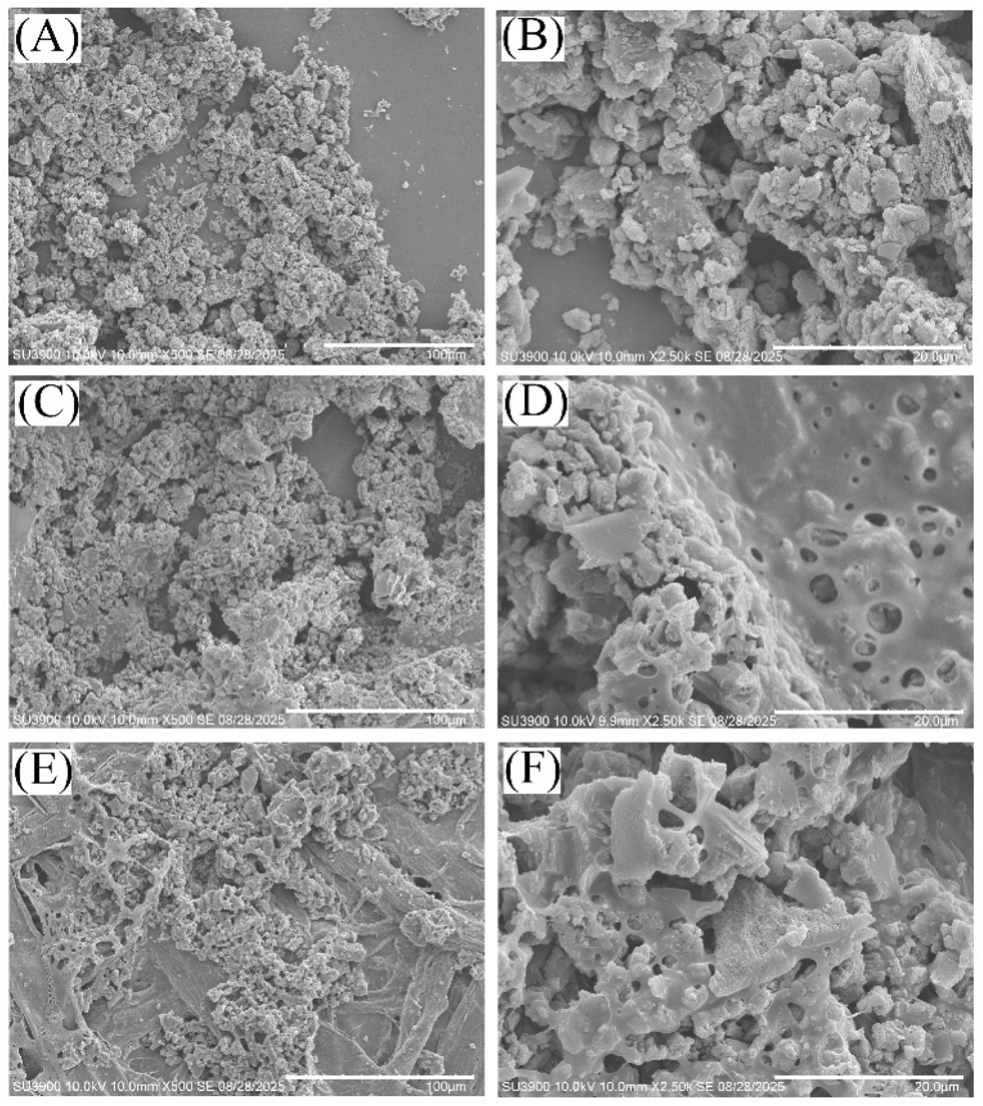
Scanning electron microscopy (SEM) images in 500× and 2500× magnification for (A and B) g-C_3_N_4_ powder on silicon, (C and D) g-C_3_N_4_ co-deposited with PIM-1 (5 : 1 weight ratio) on silicon, and (E and F) g-C_3_N_4_ co-deposited with PIM-1 on filter paper substrate.

Fluoride concentrations were determined after pH adjustment to pH 7. Fig. S1 shows measured probe potential data as a function of fluoride concentration, and the linear calibration plot is shown in Fig. S1B. The probe was re-calibrated before each measurement.

## Heptadecafluoro-1-nonanol (HDFN) degradation with g-C_3_N_4_

**Fig. 4:**
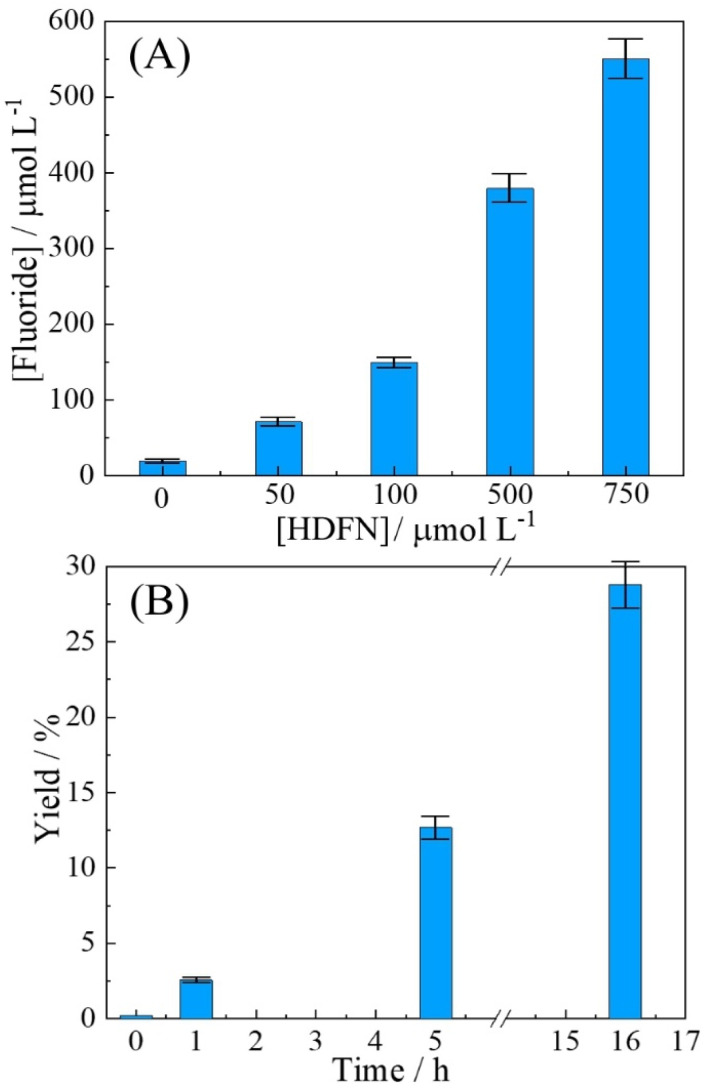
(A) Plot for photochemical HDFN degradation with 10 mg g-C_3_N_4_ suspended in 10 mL phosphate buffer pH 12 for 0–750 µmol per L HDFN (with magnetic agitation for 1 h). (B) Plot for 500 µmol per L HDFN degradation with time using 10 mg g-C_3_N_4_ suspended/agitated and 10 mL phosphate buffer pH 12 (with magnetic agitation). The determination of fluoride was performed with the pH adjusted to 7. Yield calculated based on 17 F^−^ per HDFN molecule (errors estimated ±5%).

Heptadecafluoro-1-nonanol was chosen as a model for the degradation. Initially, the evaluation of HDFN degradation with g-C_3_N_4_ as a photocatalyst was conducted in a phosphate buffer at pH 12 using various concentrations and compared based on the analytical signal of the fluoride. It was observed that the degradation of HDFN occurred at different concentrations, with a more rapid increase in fluoride production at higher concentrations (Fig. 4A). Since the fluoride probe could not function at pH 12, the analytical signal for fluoride was always determined at pH 7 after pH adjustment.

With a suspended g-C_3_N_4_ photocatalyst, the 1 h degradation of the concentrations of 50 and 100 µmol per L HDFN can be seen to yield 78 and 156 µM of fluoride, respectively (Fig. 4A). This corresponds to nearly 9.1 and 9.2% of the total fluoride yield, which is promising. Next, the time dependence of fluoride production was investigated for 500 µM HDFN (Fig. 4B). Over time, the conversion/degradation continues and after 16 h of light exposure, close to 30% yield based on total fluoride is observed. Next, the effect of immobilising the photocatalyst with a polymer of intrinsic microporosity is studied.

## Heptadecafluoro-1-nonanol (HDFN) degradation with g-C_3_N_4_ embedded into an intrinsically microporous polymer (PIM-1)

**Fig. 5:**
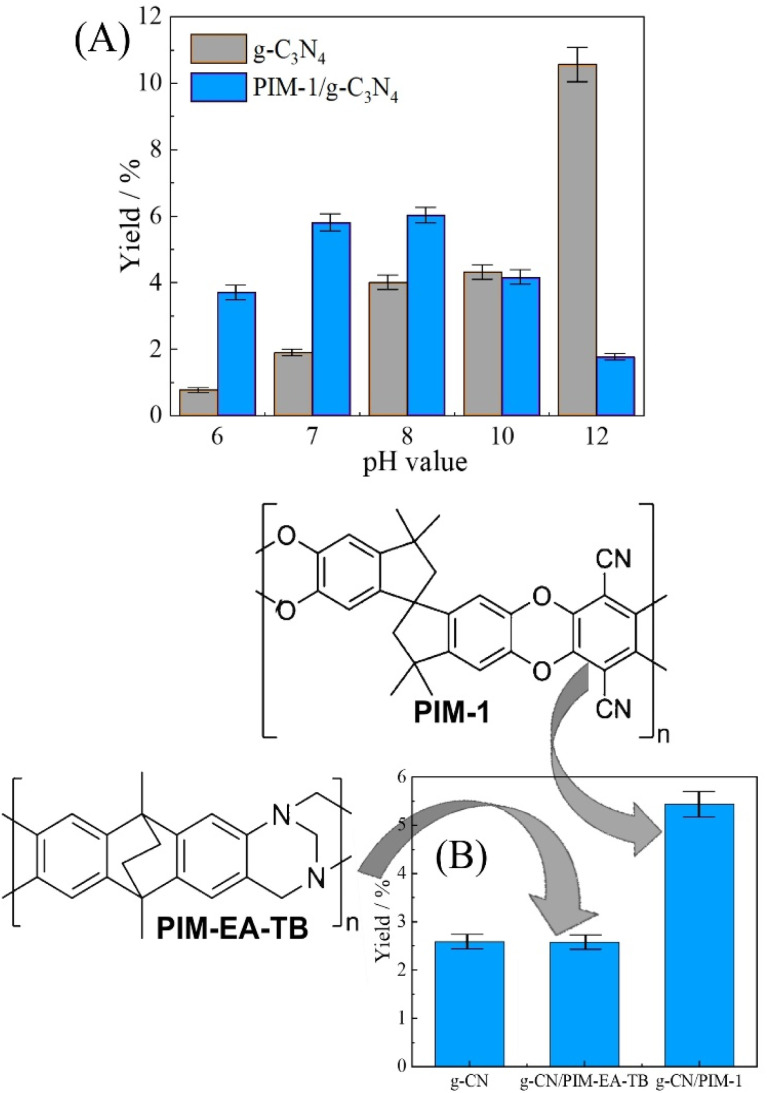
(A) 100 µmol per L HDFN degradation using 10 mg g-C_3_N_4_ suspended as powder or 10 mg g-C_3_N_4_ with 1.0 mg PIM-1 co-immobilised onto 2 × 2 cm^2^ filter paper and immersed in 10 mL phosphate buffer pH 6, 7, 8, 10, and 12 for 4 h with magnetic agitation, and posterior, determination of fluoride with pH and volume adjusted to 7 and 20 mL, respectively. (B) 100 µmol per L HDFN degradation using 10 mg g-C_3_N_4_, 10 mg g-C_3_N_4_ with 1.0 mg PIM-EA-TB in 2 × 2 cm^2^ filter paper, and 10 mg g-C_3_N_4_ with 1.0 mg PIM-1 in 2 × 2 cm^2^ filter paper and 20 mL phosphate buffer pH 7 with magnetic agitation, and posterior, determination of fluoride (errors estimated ±5%).

The effect of the pH value on the HDFN degradation rate was examined first using suspended g-C_3_N_4_ and varying pH in a phosphate buffer solution. Fig. 5A demonstrates that the use of g-C_3_N_4_ at pH 12 the is most effective, and fluoride yields reach nearly 10% after 4 h of reaction. Alkaline conditions are likely to lead to negatively charged intermediates that are easier to photo-oxidise. In the presence of a buffer solution with a lower pH, degradation occurred at a lower rate.

To evaluate the possibility of immobilizing the photocatalyst into microporous polymer, a cellulose filter paper was employed as a substrate with g-C_3_N_4_ and with PIM-1 immobilised. Fig. 5A shows data indicating that with a PIM-1 coating, photocatalysis is enhanced for pH 6, 7, and 8. This enhancement is tentatively attributed here to the accumulation of HDFN molecules within the hydrophobic and porous PIM-1 host. A test with energy-dispersive X-ray spectroscopy (EDS) has been carried out in 0.1 M phosphate buffer at pH 7 and with varying HDFN concentration. PIM-1 films (5 to 10 µm thickness) were immersed for 30 minutes, then rinsed with pure water and dried. Data in Table S1 and Fig. S2 suggest systematic uptake of HDFN into PIM-1, although not with a simple isotherm trend. This is likely due to EDS probing the bulk and the rate of HDFN transport in PIM-1 being limited.

Data in Fig. 5A suggests lower performance for HDFN reacting at g-C_3_N_4_ in PIM-1. At higher pH the formation of more hydrophilic anions/complexes might lower the uptake of HDFN into the microporous host. Enhanced HDFN degradation, compared to suspended g-C_3_N_4_, is particularly interesting at neutral pH conditions. A comparison of two types of intrinsically microporous host materials was attempted for (i) PIM-1 and (ii) PIM-EA-TB; see Fig. 5B. Clearly, the more hydrophobic PIM-1 material is more effective. Repeatability by changing to a fresh solution was tested for the degradation of 500 µmol per L HDFN solution using 10 mg g-C_3_N_4_ at pH values of 7.0 and 12.0, obtaining (for 3 repeats) 0.9 ± 0.3% and 7.8 ± 0.8%, respectively. More generally, errors in these experiments (typically ±5% RSD) are associated with g-C_3_N_4_ batch and positioning of the light source.

The distance from the LED light source can directly influence the HDFN degradation rate due to variation in the LED power with distance. Hence, it was evaluated at a 6.0 and 4.0 cm distance using g-C_3_N_4_ without (suspended) and with immobilization employing PIM-1. As expected, decreasing the distance promoted an increase in the degradation of this molecule, yielding 1.90 and 5.82% yields for g-C_3_N_4_ and PIM-1/g-C_3_N_4_, respectively, at 6.0 cm (power approx. 14 mW cm^−2^), and 4.16 and 9.45% yields for g-C_3_N_4_ and PIM-1/g-C_3_N_4_, respectively, at 4.0 cm (power approx. 27 mW cm^−2^). This distance of 4.0 cm was chosen for the next experiments.

## Heptadecafluoro-1-nonanol (HDFN) degradation with g-C_3_N_4_: light intensity and catalyst recycling

**Fig. 6:**
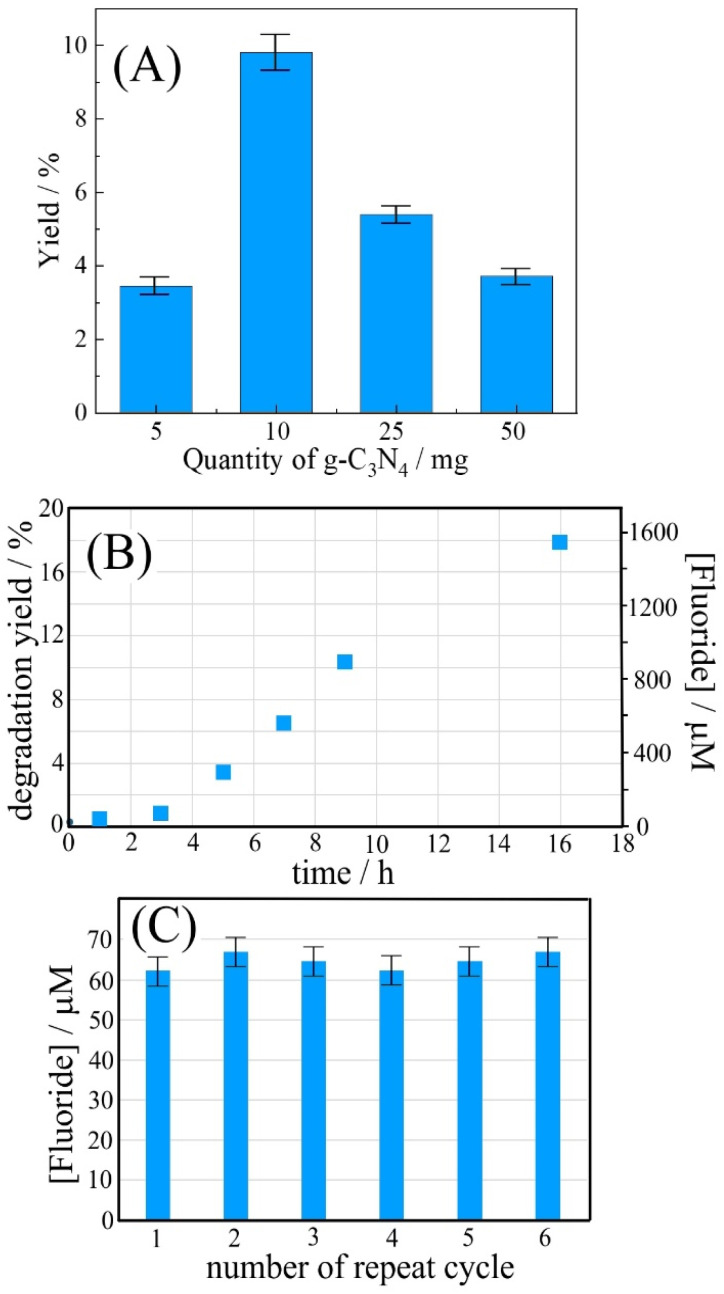
(A) Fluoride yield for 100 µmol per L HDFN degradation over 4 h time using 5–50 mg g-C_3_N_4_ (coated with 1.0 mg PIM-1 onto a 2 × 2 cm^2^ filter paper) immersed in 20 mL phosphate buffer pH 7 with magnetic agitation. (B) Plot of fluoride production and degradation yield *versus* time for 10 mg g-C_3_N_4_ (coated with 1.0 mg PIM-1 onto a 2 × 2 cm^2^ filter paper) immersed in 20 mL phosphate buffer pH 7 with 100 mM HDFN. (C) Bar graph for repeat measurements using the same catalyst impregnated filter paper repeatedly (2 h; 100 mM HDFN; 20 mL phosphate buffer pH 7) (errors estimated ±5%).

The quantity of g-C_3_N_4_, ranging from 5 to 50 mg (over 2 × 2 cm^2^), immobilized with PIM-1 onto the filter paper, was evaluated to further increase the rate of HDFN degradation. The results in Fig. 6A demonstrate that an increased g-C_3_N_4_ photocatalyst amount can lead to a decrease in yield. This is likely to be due to thicker catalyst/PIM-1 deposits causing restricted transport of reagents and products. HDFN would have to diffuse *via* micropores into the photoactive film, which will severely limit the rate of photodegradation in thicker deposits. A further problem could be associated with thicker films gradually blocking some of the light. Optimum conditions were obtained using 10 mg g-C_3_N_4_. Therefore, 10 mg was chosen for further experiments.

The time dependence of HDFN degradation was examined for 100 mM HDFN and the immobilised g-C_3_N_4_ photocatalyst. Similar to the case of suspended g-C_3_N_4_ (Fig. 4B), the production of fluoride continues with time, but with an onset delay in the first 2 h (due to H-atoms on the first carbon, Fig. 1C). The repeat use of the g-C_3_N_4_/PIM-1 photocatalyst on filter paper was investigated for 2 h exposure and fresh 100 µM HDFN solution in each repeat cycle (Fig. 6C). The catalyst retains photo–degradation activity and can therefore be re-used and recovered.

## Conclusion

It has been shown that the photodegradation of heptadecafluoro-1-nonanol (HDFN) with g-C_3_N_4_ is pH-dependent and more effective in aqueous alkaline environments. Importantly, the use of a PIM-1 microporous polymer as a host environment substantially increases the rate of HDFN degradation even at pH 7 in a phosphate buffer solution. This is tentatively assigned to the accumulation of heptadecafluoro-1-nonanol into the hydrophobic micropores close to the photocatalyst surface. A full study of the adsorption isotherm for HDFN or similar PFAS materials in PFAS will be desirable in the future. The molecularly rigid nature of PIM-1 prevents/suppresses the direct photocatalytic degradation of the host polymer, and it enhances the degradation of the HDFN guest in the microporous environment. The molecularly rigid PIM-1 cannot interact with the photocatalyst surface and therefore maintains catalyst activity.

In the future, the nature of degradation intermediates will have to be assessed and monitored in detail including detection of H_2_O_2_. More generally, longer term performance testing and catalyst re-use need to be investigated in more detail. New in *operando* experimental tool to follow the in/out flow of the fluorine/fluoride will be desirable. The role of composite geometry, *i.e*. the active zone during photocatalysis, needs more attention. The beneficial effect of PIMs and similar microporous materials applied to photocatalysts should be studied and developed more systematically. Initial accumulation of substrates and turnover of reaction intermediates will be affected, and more hydrophobic (potentially also more toxic) intermediates will be potentially retained and destroyed more effectively.

## Author contributions

Fernanda C. O. L. Martins: data curation, formal analysis, investigation, writing – original draft and review & editing; Wanessa R. Melchert: conceptualization, funding acquisition, supervision, writing – review & editing; Akalya Karunakaran: data curation, formal analysis, investigation, methodology, writing – review & editing; Chris R. Bowen: supervision, writing – review & editing; Nicholas Garrod: investigation, data curation; Philip J. Fletcher: investigation, data curation; Mariolino Carta: methodology, supervision, writing – review & editing; Dominic Taylor: methodology, resources, writing – review & editing; Neil B. McKeown: conceptualization, funding acquisition, supervision, writing – review & editing; Frank Marken: conceptualization, formal analysis, methodology, resources, supervision, writing – original draft and review & editing.

## Conflicts of interest

There are no conflicts to declare.

## Supplementary Material

RA-016-D5RA07284K-s001

## Data Availability

The data supporting this article have been included as part of the main document and the supplementary information (SI). Supplementary information is available. See DOI: https://doi.org/10.1039/d5ra07284k.
