# Supplementary material for: Intrinsically microporous polymer (PIM-1) enhanced degradation of heptadecafluoro-1-nonanol at graphitic carbon nitride (g-C3N4)
Source: RSC Adv. 2026 Jan 2;16(1):28–33. doi: 10.1039/d5ra07284k (PMC12757717; doi:10.1039/d5ra07284k)
Supplement: RA-016-D5RA07284K-s001 [file RA-016-D5RA07284K-s001.pdf]

Supplementary Information (SI) for RSC Advances.
This journal is © The Royal Society of Chemistry 2025

SUPPORTING INFORMATION

Intrinsically Microporous Polymer (PIM-1) Enhanced Degradation of Heptadecafluoro-1-nonanol at Graphitic Carbon Nitride (g-C_3N_4)

Fernanda C.O.L. Martins,[a,b] Wanessa R. Melchert,[c] Akalya Karunakaran,[a,d] Chris R. Bowen,[d] Nicholas Garrod,[a] Philip J. Fletcher,[e] Mariolino Carta,[f] Dominic Taylor,[g] Neil B. McKeown,[g] and Frank Marken*[a]

[a] *Department of Chemistry, University of Bath, Claverton Down, Bath, BA2 7AY, UK*

[b] *Center for Nuclear Energy in Agriculture, University of São Paulo, P.O. Box 96, Piracicaba, SP 13400-970, Brazil*

[c] *College of Agriculture Luiz de Queiroz, University of São Paulo, P.O. Box 9, Piracicaba, SP 13418-970, Brazil*

[d] *Department of Mechanical Engineering, University of Bath, BA2 7AY, Bath, UK*

[e] *University of Bath, Imaging Facility, Bath, BA2 7AY, UK*

[f] *Department of Chemistry, Swansea University, College of Science, Grove Building, Singleton Park, Swansea SA2 8PP, UK*

[g] *EaStCHEM, School of Chemistry, University of Edinburgh, Joseph Black Building, David Brewster Road, Edinburgh, Scotland EH9 3JF, UK*

CONTENT

Experimental 2

Instrumentation 2

Figure S1 3

Table S1 3

Figure S2 4

Table S2 4

Table S3 5

Table S4 5

Table S5 6

Table S6 6

Table S7 7

Table S8 7

References 8

Experimental. Heptadecafluorononanol (2,2,3,3,4,4,5,5,6,6,7,7,8,8,9,9,9-heptadecafluoro-1-nonanol, Sigma-Aldrich, 446823) and all other reagents were purchased and used without further purification. Polymers of intrinsic microporosity were synthesised with molecular weight >70 kD following literature methods for PIM-1[1] and for PIM-EA-TB[2]. The photocatalyst g-C_3N_4 was synthesized following a literature recipe[3] using 5 g of melamine within a ceramic boat with a lid, which was placed into a tube furnace with a temperature ramp to 500 °C, where the temperature was maintained for 4 h.

Instrumentation. Fluoride release was quantified with a potentiometric fluoride probe FC301B (Hanna Instruments, US). Chronopotentiometric analyses were performed with a potentiostat/galvanostat from Metrohm-Eco Chemie model µAUTOLB III with NOVA 2.1.2 software (Metrohm-Eco Chemie, NL). Zero current potentiometry was performed versus a saturated calomel electrode (SCE). For pH measurements, a commercial glass membrane pH-probe (Voltcraft 127752) was employed. Photochemical processes were performed with a light emitting diode (LED) light source (λ = 385 nm, approx. 27 mW cm^{-2} at 4 cm distance, Thorlabs Ltd.). A water purification system from CE Instruments Ltd. was used to obtain purified water with resistivity not lower than 18.2 Ωcm at 20 °C. Scanning electron microscopy (SEM) and energy-dispersive X-ray spectroscopy (EDS) was carried out on using a Hitachi SU3900 system with an Oxford Instruments X-Max 170 mm^2 EDS detector.

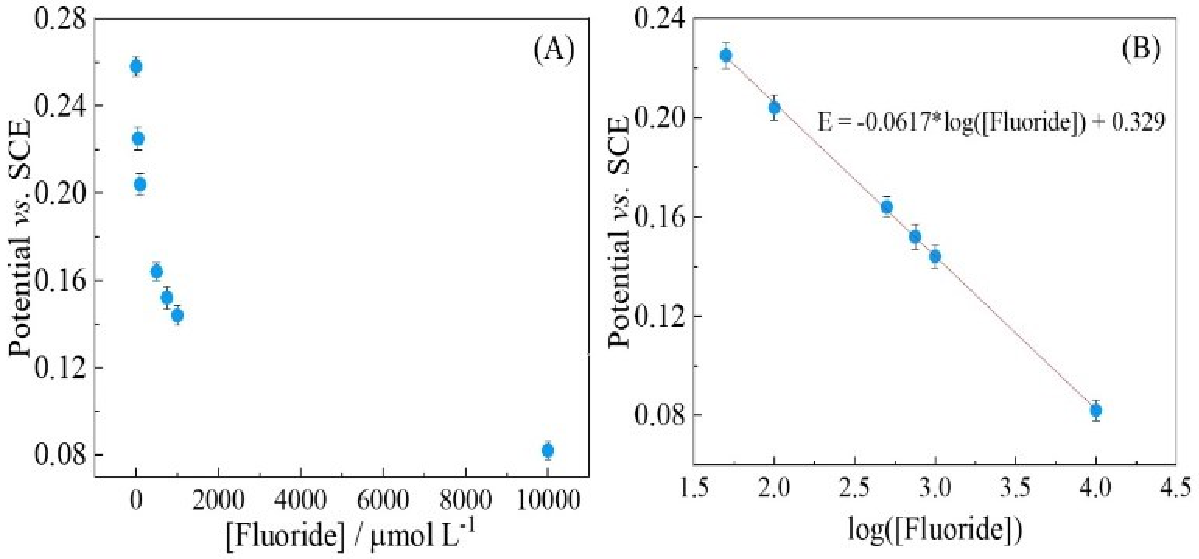

Figure S1. Plot of potential *versus* (A) fluoride concentration and (B) log(fluoride concentration) for the bare fluoride-probe in 0.10 mol L^{-1} phosphate buffer pH 7. The sensor calibration was repeated/verified daily to maintain accuracy.

Table S1. Energy dispersive x-ray spectroscopy data (5 to 10 µm thick PIM-1 films on silicon wafers; area 5 mm × 5 mm; treated by immersion for 30 mins into HDFN solution in 0.1 M phosphate buffer at pH 7; rinsed with water; dried; 5 kV electron beam) as a function of HDFN concentration.

		**atom%**					**ratios of atom%**		
#	C(HDFN) /mol L^{-1}	C	N	O	F	Σ(Na, Al, Si, P, K)	N/C [a]	N/O [b]	F/N
1	2.0 E-04	71.6	6.74	15.5	5.2	0.92	0.094	0.43	0.77
2	2.0 E-05	73.6	6.84	15.2	0.1	4.21	0.093	0.45	0.015
3	2.0 E-06	75.3	7.27	16.3	0.09	1.06	0.097	0.44	0.012
4	2.0 E-07	75.3	6.99	16.3	0.1	1.34	0.093	0.43	0.014
5	2.0 E-08	73.7	7.02	15.8	0.04	3.36	0.095	0.44	0.0057
6	2.0 E-09	73.2	6.75	14.9	0.05	5.07	0.092	0.45	0.0074
7	0	75.7	6.29	15.9	0.0	2.07	0.083	0.40	0.0

[a] anticipated for pure PIM-1 ($C_{29}H_{20}O_4N_2$) is a ratio of 0.069.

[b] anticipated for pure PIM-1 is a ratio of 0.50.

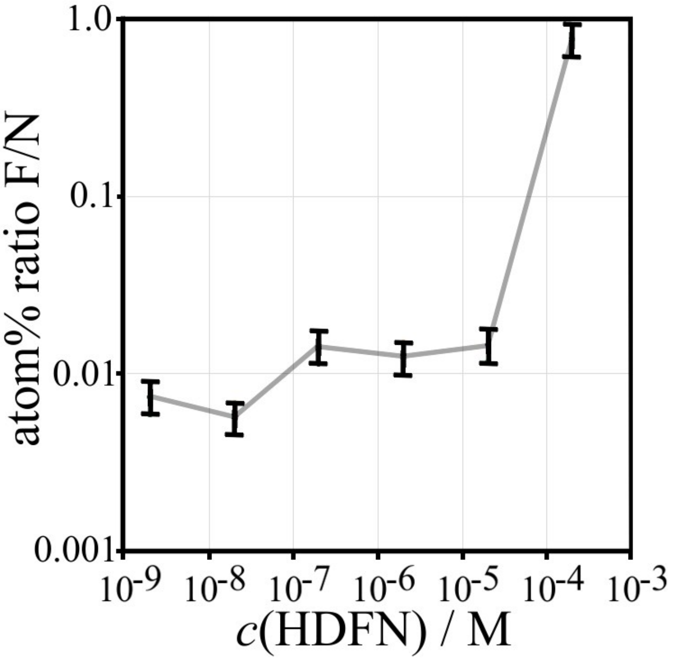

Figure S2. Plot energy dispersive x-ray spectroscopy data (F/N ratio in Table S1; 5 to 10 µm thick PIM-1 films on silicon wafers; area 5 mm × 5 mm; immersed for 30 mins into HDFN solution in 0.1 M phosphate buffer at pH 7; rinsed with water; dried; 5 kV electron beam) as a function of HDFN concentration. Error estimated bars based on ±10% error in atom% readings.

Table S2 (Figure 4A). Fluoride concentration from photochemical HDFN degradation with 10 mg g-C_3N_4 suspended in 10 mL phosphate buffer pH 12 for 0 – 750 µmol L^{-1} HDFN (with magnetic agitation for 1 h). The determination of fluoride was performed with the pH adjusted to 7. Yield calculated based on 17 F- per HDFN molecule (errors estimated ±5%).

**PFAS concentration / µM**	**Fluoride concentration / µM**
0	19.14 ± 0.96
50	70.68 ± 3.53
100	149.08 ±7.45
500	378.97 ± 18.95
750	550.40 ± 27.52

Table S3 (Figure 4B). Fluoride yield for 500 µmol L^{-1} HDFN degradation with time using 10 mg g-C_3N_4 suspended/agitated and 10 mL phosphate buffer pH 12 (with magnetic agitation), with evaluation of degradation time. The determination of fluoride was performed with the pH adjusted to 7. Yield calculated based on 17 F- per HDFN molecule (errors estimated ±5%).

**Degradation time / h**	**Yield / %**
0	0.19 ± 0.01
1	2.55 ± 0.13
5	12.68 ± 0.63
16	28.81 ± 1.44

Table S4 (Figure 5A). Fluoride yield for 100 µmol L^{-1} HDFN degradation using (i) 10 mg g-C_3N_4 suspended or (ii) 10 mg g-C_3N_4 1.0 mg PIM-1 immobilised onto 2 × 2 cm^2 filter paper (Whatman 1) and immersed in 10 mL phosphate buffer pH 6, 7, 8, 10, and 12 for 4 h with magnetic agitation, and posterior, determination of fluoride with pH and volume adjusted to 7 and 20 mL, respectively (errors estimated ±5%).

**pH value**	**(i) Yield / %**	**(ii) Yield / %**
6	0.78 ± 0.04	3.72 ± 0.19
7	1.90 ± 0.09	5.82 ± 0.29
8	4.01 ± 0.20	6.04 ± 0.30
10	4.32 ± 0.22	4.16 ± 0.21
12	10.57 ± 0.53	1.76 ± 0.09

Table S5 (Figure 5B). Fluoride yield for 100 µmol L^{-1} HDFN degradation using 10 mg g-C_3N_4, 10 mg g-C_3N_4 with 1.0 mg PIM-EA-TB in 2 × 2 cm^2 filter paper, and 10 mg g-C_3N_4 with 1.0 mg PIM-1 in 2 × 2 cm^2 filter paper and 20 mL phosphate buffer pH 7 with magnetic agitation, and posterior, determination of fluoride (errors estimated ±5%).

**Evaluation of immobilization**	**Yield / %**
g-C_3N_4	2.56 ± 0.13
g-C_3N_4/PIM-EA-TB	2.76 ± 0.14
g-C_3N_4/PIM-1	5.40 ± 0.27

Table S6 (Figure 6A). Fluoride yield for 100 µmol L^{-1} HDFN degradation over 4 h time using 5 – 50 mg g-C_3N_4 (coated with 1.0 mg PIM-1 onto a 2 × 2 cm^2 filter paper) immersed in 20 mL phosphate buffer pH 7 with magnetic agitation (errors estimated ±5%).

**Quantity of g-C_3N_4 / mg**	**Yield / %**
5	3.45 ± 0.17
10	9.81 ± 0.49
25	5.40 ± 0.27
50	3.72 ± 0.19

Table S7 (Figure 6B). Fluoride production and degradation yield versus time for 10 mg g-C_3N_4 (coated with 1.0 mg PIM-1 onto a 2 × 2 cm^2 filter paper) immersed in 20 mL phosphate buffer pH 7 with 100 µM HDFN (errors estimated ±5%).

**Time / h**	**Potential / V vs. SCE**	**Log(F^-/M)**	**[F^-]/ µM**	**Yield / %**
0	0.184	1.449	28.1 ± 1.4	0.33 ± 0.02
1	0.172	1.638	43.4 ± 2.2	0.51 ± 0.03
3	0.158	1.858	72.2 ± 3.6	0.85 ± 0.04
5	0.120	2.457	286.2 ± 14.3	3.37 ± 0.17
7	0.102	2.740	549.7 ± 27.5	6.47 ± 0.32
9	0.089	2.945	880.8 ± 44.1	10.36 ± 0.52
16	0.074	3.181	1517.4 ± 76.0	17.85 ± 0.89

Table S8 (Figure 6C). Fluoride production for repeat measurements using the same catalyst impregnated filter paper repeatedly (2 h; 100 µM HDFN; 20 mL phosphate buffer pH 7) (errors estimated ±5%).

**Experiment**	**Potential / V vs. SCE**	**Log(F^-/M)**	**[F^-]/ µM**
1	0.162	1.795	62.4 ± 3.1
2	0.160	1.827	67.1 ± 3.4
3	0.161	1.811	64.7 ± 3.2
4	0.162	1.795	62.4 ± 3.1
5	0.161	1.811	64.7 ± 3.3
6	0.160	1.827	67.1 ± 3.4

References

[1] P.M. Budd, E.S. Elabas, B.S. Ghanem, S. Makhseed, N.B. McKeown, K.J. Msayib, C.E. Tattershall, D. Wang, *Adv. Mater.*, 2004, **16**, 456–459.

[2] M. Carta, R. Malpass-Evans, M. Croad, Y. Rogan, J.C. Jansen, P. Bernardo, F. Bazzarelli, N.B. McKeown, *Science*, 2013, **339**, 303–307.

[3] A. Karunakaran, K.J. Francis, C.R. Bowen, R.J. Ball, Y.Z. Zhao, L.N. Wang, N.B. McKeown, M. Carta, P.J. Fletcher, R. Castaing, M.A. Isaacs, L.J. Hardwick, G. Cabello, I.V. Sazanovich, F. Marken, *Chem. Commun.*, 2023, **59**, 7423–7426.
